# Selective laser trabeculoplasty (SLT) performed by optometrists—enablers and barriers to a shift in service delivery

**DOI:** 10.1038/s41433-021-01746-0

**Published:** 2021-08-13

**Authors:** Evgenia Konstantakopoulou, Lee Jones, Neil Nathwani, Gus Gazzard

**Affiliations:** 1 NIHR Biomedical Research Centre at Moorfields Eye Hospital NHS Foundation Trust, London, UK; 2 Institute of Ophthalmology, University College London, London, UK; 3 Division of Optics and Optometry, University of West Attica, Athens, Greece

**Keywords:** Health services, Glaucoma

## Abstract

**Background/objectives:**

To explore the acceptability, training requirements, enablers and barriers of optometrist-delivered SLT.

**Subjects/methods:**

Optometrists, fellowship and consultant grade ophthalmologists, hospital managers and patients were interviewed using pre-defined topic guides. Interviews were audio-recorded, transcribed, and subjected to thematic analysis. Overarching themes were defined by the study aims and the topic guides; subthemes were derived from the interview data.

**Results:**

Sixty-six participants (three managers, eight glaucoma specialist consultant ophthalmologists, seven clinical glaucoma fellows, 12 optometrists (two of them performing SLT), two ophthalmic nurses and 34 patients) participated in the study. Overarching themes (and subthemes) were: necessity of non-medical SLT delivery, clinical practice and training, advantages, disadvantages, concerns, challenges, community delivery of SLT, patient values and other healthcare professionals that could also deliver SLT.

**Conclusions:**

Certain clinical pre-requisites, such as gonioscopy and independent prescribing rights, were perceived as necessary for undertaking SLT training. An optometrist-delivered SLT service was expected to benefit the NHS, but there was an identified need of a standardised training scheme and robust governance. Patients were accepting of an optometrist-delivered SLT service in the hospital eye service.

## Introduction

Hospital optometrists routinely, often independently, assess and manage patients with glaucoma in the Hospital Eye Service (HES), as a means to address an imbalance between capacity and demand in the glaucoma service nationwide.

Selective laser trabeculoplasty (SLT) is an outpatient procedure for lowering intra-ocular pressure (IOP), equally effective to eye drops; patients may maintain freedom from eye drops for at least 3 years and experience fewer ocular adverse events compared to treatment with eye drops [1]. Between 2019 and 2020 there was a significant increase (33%) in SLT procedures performed in the HES, reaching the highest number of annual attendances in the last 10 years [2].

Non-medical delivery of SLT is taking place in an ad-hoc basis in the UK [3], but evidence on service efficacy, clinical safety or cost-effectiveness is limited [4]. Despite the national need to expand glaucoma care to non-ophthalmologists, limited peer-reviewed data [4, 5] are hindering the process of expanding SLT delivery to optometrists. This study aimed to explore if optometrist-delivered SLT is acceptable and of value to support future implementation across the UK, to gather opinions on training requirements and to understand enablers and barriers to a change in service delivery.

## Methods

### Recruitment

Clinicians and hospital managers were members of staff at Moorfields Eye Hospital NHS Foundation Trust; patients were recruited from the Moorfields SLT clinics and from the LiGHT trial cohort [1, 6] and had undergone SLT at least once, either by a consultant, a clinical fellow or an optometrist.

Participants were provided with an information sheet explaining the aims of the study and a consent form and were given ample time to consider their participation. The study was approved by the Health and Care Research Wales Ethics Committee (20/SW/0033) and was conducted according to the tenets of the Declaration of Helsinki. Written, informed consent was obtained from participants. All participants’ information was anonymised using a study ID, before being entered into a computer database.

### Data collection

Semi-structured interviews were conducted and audio-recorded between April and December 2020. Median (IQR) interview duration was 12 min (8–17). Interviews were based on a topic guide (Table 1), which was developed before the commencement of the study, after consultation with clinicians and experts in qualitative research. The topic guide was piloted in a small number of initial interviews and adapted based on feedback. Questions were open-ended, promoting detailed responses and requiring minimum facilitator input. An attempt was made not to ask leading questions although, often, explanation of professional roles within the HES was required for patients. Prompts were used to encourage participant elaboration of a specific point. Prior to the interview, it was made clear to participants that no particular answers were expected and that their views could be expressed freely. All participants were given the opportunity to add any discussion points they considered relevant at the end of the interview.

**Table 1:** Topic guides used for interviewing patients, clinicians and managers.

Patients	Clinicians	Managers
• Can you tell me about your experience of having laser therapy?	• What are your thoughts about optometrists delivering SLT procedures?	• What are your thoughts about optometrists delivering SLT procedures?
• What matters to you, in terms of the person who performs the procedure?	• What would you consider to be the necessary training and assessment that optometrists should undergo before performing SLT?	• What advantages or disadvantages, if any, can you see for the hospital having optometrists perform SLT procedures? How would this affect the service you are managing?
• Who would you expect to perform this type of procedure? Why?	• How many supervised/observed eyes do you think should be treated by an optometrist/non-medical professional before they are signed off as competent?	• Is it necessary that optometrists, and not only ophthalmologists, perform SLT in the future?
• How would you feel about a health professional who is not medically trained, but has undergone appropriate laser training courses, performing your procedure?	• There’s an argument that optometrists should be working in consultant-led hospital glaucoma clinics in order to train for SLT. Would you agree or disagree and why? Could this be open to community optometrists?	• What challenges, if any, do you think institutions might face when setting up an SLT service that is delivered by optometrists?
• Can you see any advantages to yourself or the hospital having laser therapy performed by a health professional who is not medically trained?	• Should there be any regular audit process to ensure the treatment is done accurately and the outcomes are similar to other colleagues? If yes, how might this be done, and how often?	• It is possible that, in the future, optometrists will routinely perform laser therapy instead of ophthalmologists. What do you think is the most important thing health services should consider before this becomes a reality
• Can you see any disadvantages to yourself or the hospital having laser therapy performed by a health professional who is not medically trained?	• Under what circumstances, if any, would you feel comfortable with optometrists performing SLT procedures?	
• You’ve previously had laser therapy performed by a doctor/someone who is not a doctor. If you were to have this procedure again, would you feel differently if this time the procedure was done by someone who is not a doctor/a doctor?	• What advantages/disadvantages, if any, can you see for the hospital having optometrists performing SLT procedures?	
	• Is it necessary that optometrists, and not only ophthalmologists, perform SLT?	
	• How appealing is the opportunity to undertake specialised training in delivering SLT? *Optometrists only*	
	• What do you believe to be the main motivations for optometrists wanting to take on the role of these procedures?	

Due to the COVID-19 pandemic interviews were conducted remotely via telephone or online communication platforms by two experienced research fellows (EK; female and LJ; male). The study followed the Consolidated Criteria for Reporting Qualitative Research (COREQ) [7] (Appendix 1).

### Analysis

Data were analysed by two researchers independently using thematic analysis [8]. Interview transcripts were read and reread, themes were identified and quotes were allocated to each theme. Overarching themes were defined by the study aims and the topic guide; subthemes were inductively derived from the interview data. Any differences between the analyses of the two researchers were discussed until a consensus was reached. The qualitative software package NVIVO V.11 (QSR International, Cambridge, Massachusetts, USA) was used for data management. Data saturation was determined post-hoc and indicated further data collection or analysis was unnecessary. To assess the emerging themes and the validity of the findings, participant validation of the results was conducted with four interviewees.

### Participants

A total of 66 participants were recruited; three managers, eight glaucoma specialist consultant ophthalmologists, seven clinical glaucoma fellows, 12 optometrists (two regularly performing SLT), two ophthalmic nurses and 34 patients.

Purposive sampling was used where patient participants were invited to the interview by their treating clinician while attending regular follow-up visits for assessment of their eye health, or from a database of participants receiving SLT in the LiGHT trial. A total of 15 patients were recruited from the LiGHT trial cohort and 19 patients were recruited from the NHS SLT clinics; in total 15 of the patients had undergone SLT by an ophthalmologist, 17 had undergone SLT by an Optometrist and two patients had undergone SLT by both an ophthalmologist and an optometrist. All patients recruited from the LiGHT trial cohort had received SLT by an ophthalmologist; two of them had also received SLT by an Optometrist, as part of the NHS SLT clinics. Of the patients recruited for the NHS clinics, two had SLT performed by an ophthalmologist and 17 by an Optometrist. The researchers corresponded with participants via email and telephone during recruitment and had met some participants previously through the LiGHT trial.

Data were coded into overarching themes relating to:

- The necessity of an optometrist-delivered SLT service.
- The optometrists’ clinical practice/training.
- Advantages, disadvantages and challenges.
- Patients’ expectations.
- Other non-medical professionals that could deliver SLT.

The identified themes are presented using quotes from the transcripts, in italicised font, followed by the role of the person providing the quote. Additional quotes are available in Appendix 2.

## Results

### Necessity of non-medical professionals performing SLT

Non-medical delivered SLT was considered a necessity for large glaucoma departments by clinicians and managers, due to growing backlogs:

*We do need to increase our capacity and we don’t have the medical workforce to do that. So an optometry platform makes a lot of sense* (Consultant).

However, it was noted that the extent of this urgency may vary for smaller departments.

### Clinical practice and training

#### Non-medical professions

Optometrists were considered the most appropriate non-medically trained professionals to perform SLT, due to their background training and their clinical skills. Most patients showed a preference for optometrists, related to their familiarity with optometrists and experience of decision making.

*Often an optometrist has looked at my eyes and has made an assessment, based on other aspects of the treatment and its result. That knowledge cannot be held by just a health professional* (Patient).

#### Professional activity prior to and around SLT training

Prior exposure of optometrists to consultant-led glaucoma clinics was considered necessary.

*One can only really understand the clinical importance of the treatment and the follow-up care when some of it is within a supervised consultant environment* (Consultant).

Performing SLT in an environment with consultant presence was also considered important for providing clinical support and was valued by patients.

*If you were going to change the qualification of the person carrying out the procedure, you need to make sure that they have access, on the spot, to consultant advice, should something either go wrong or there be complications or the need for additional advice* (Patient).

#### Skills prior to SLT training

Proficiency in gonioscopy and being qualified to independently prescribe medication were considered necessary skills for commencing SLT training. The Higher Certificate in Glaucoma was also suggested as useful for optometrists performing SLT.

*If optometrists don’t have the skills to assess an angle themselves, the patient might end up worse than they were before* (Consultant).

#### SLT training

There was a consensus that the SLT training of optometrists should commence with lectures on laser safety, risks, adverse events and management. This would be followed by a period of observation and delivering SLT under supervision. There was no agreement on the number of supervised eyes an optometrist would have to treat before delivering SLT independently, ranging from 5 to 100, and the variability of training needs was acknowledged.

*There needs to be a sort of self-assessment; do they actually feel comfortable doing the procedure independently?* (Consultant).

Clinicians also considered the standardisation and governance of training schemes important, mainly in relation to patient safety.

### Advantages

#### Capacity and reduced waiting times

Enabling optometrists to undertake training and routinely deliver SLT was considered an opportunity to increase capacity and reduce waiting times:

*It would enable us to put more patients through. There’s probably a reluctance to initiate treatment with SLT because of this perceived capacity issue and the bother of actually getting the patient onto the laser and doing the procedure. I think it would remove a barrier to some extent* (Consultant).

There was also recognition that diversifying the workforce would help alleviate future demand for SLT, allowing improved clinic scheduling, whereby dedicated SLT clinics could be established to facilitate greater patient throughput.

#### Costs

Clinicians believed that implementation of an optometrist SLT service could offer cost savings to the NHS. The extent to which an optometrist service could be economically valuable would dependent on current clinical service arrangements; optometrists replacing consultants would offer the greatest cost differential.

#### Optometrist availability/stability/manpower

The stability of the optometry profession was seen as an advantage. Unlike medical personnel, optometrists were considered likely to remain at the same unit for longer periods of time, enabling services to provide more continuity of care.

*Once you’ve trained optometrists up, they tend to stay in those positions for long periods of time, which means you’ve got continuity of care and service* (Manager and Optometrist).

#### Ophthalmologists focusing on complex cases

Clinicians identified that an inter-disciplinary approach could allow ophthalmologists to treat more complex cases and spend more time performing surgery:

*‘Glaucoma consultants are busy with advanced cases and their associates are tied up doing SLTs, so advanced cases are backlogged. Optometrists can be utilised to run those laser clinics and the associates can be at the consultant’s disposal.’* (Optometrist).

### Disadvantages and concerns

#### Time commitments

The single disadvantage recognised by clinicians and managers was the time commitments required for training, with a greater impact on smaller units.

*Somebody’s got to do the training, so that takes time from whoever’s delivering the training* (Consultant).

#### Governance and litigation

Governance was identified as a priority when setting up an optometrist-delivered SLT service within the NHS.

*Institutions may find difficulties around the lines of responsibility, where the clinical responsibility and the litigation risks lie and how it will be handled within each department. We need very specific guidance about clinical governance and crown indemnity for people performing these procedures*. (Manager & Consultant).

### Challenges

#### Professional boundaries and interactions

The development of an optometrist-delivered SLT service was considered to require close collaboration between ophthalmologists and optometrists. Concerns were raised around cultural barriers between professionals and how this could affect the successful development of the service.

*I think it’s also about breaking down the hierarchy between consultants and optometrists and whether there’s perhaps a cultural issue to overcome* (Manager).

#### Impact on ophthalmology training

Clinicians thought a negative impact on ophthalmology training was unlikely, provided there were appropriate provisions and careful planning. Inter-professional training was suggested as a means to ensure training opportunities for ophthalmologists were not limited.

*I don’t see why ophthalmologists can’t train with an optometrist who’s skilled at doing SLT* (Consultant).

It was acknowledged that issues could arise in small HES glaucoma units, but a standardised training scheme for optometrists was considered to have benefits for ophthalmologists.

*If you invest in the equipment and a training framework for optometrists then ophthalmology trainees will probably benefit, as long as they’re included in the thinking for providing their training*. (Consultant)

### Slt in the community

There was agreement that the clinical decision for SLT should be made in the HES. Most clinicians felt that a community delivery of SLT, i.e. outside a hospital setting by primary care optometrists, would need to be accompanied by regular, audited activity of the optometrist in HES glaucoma clinics.

*Unless community optometrists are working regularly in a HES glaucoma clinic, I don’t think I would feel as comfortable* (Consultant).

Patients were hesitant about SLT delivery in the community and valued the clinical support available in a hospital setting.

*I would worry if it’s a high street optometrist because I see the procedure as something that hospitals do. With a big hospital, if anything did go wrong, I have utter confidence that there would be somebody there to help* (Patient).

### Patient expectations and values

Most patients expected the treatment to be delivered by an ophthalmologist, but approved of the delivery of SLT by appropriately trained optometrists. Patients valued appropriate training and communication skills most; any professional delivering the SLT was expected to make them feel comfortable, explain the procedure and provide clear advice.

### Other healthcare professionals

Ophthalmic nurses were considered appropriate non-medical professionals to deliver SLT, albeit with different training needs and as a second stage approach. A sub-specialisation in glaucoma and/or advanced clinical skills was perceived as necessary. The views of nurse practitioners were aligned with those of optometrists and ophthalmologists around governance and impact on ophthalmology training. Patients showed some hesitation around nurses delivering SLT and identified the need for prior ophthalmic specialisation.

## Discussion

Optometrists are a specialist workforce actively involved in glaucoma management, with many achieving independent clinical practice following appropriate training. Despite the current involvement of non-medical professionals in glaucoma management, the Royal College of Ophthalmologists has proposed the introduction of yet new models of care [9, 10]. Among those, the full management of glaucoma patients by appropriately trained non-medical professionals is expected to double clinic capacity [10, 11]. SLT is a clinically- and cost-effective outpatient procedure for lowering IOP [1, 12], currently delivered by UK optometrists in an ad-hoc basis.

### Advantages of a change in service delivery

Current SLT backlogs were reported to often favour the prescription of eye drops compared to SLT. During 2012 there were over 8 million items dispensed for the lowering of IOP, resulting in a cost of over £105 million [13, 14]. SLT has been shown to allow freedom from eye drops for nearly 75% of treated patients for at least 3 years, whilst leading to fewer ocular adverse events and offering cost savings to the NHS [1, 12]. Barriers to implementing SLT in the HES may be impeding the delivery of best care, adding to current backlogs and burdening NHS expenditure.

A shift of SLT delivery to optometrists was associated with perceived cost saving, mainly due to a difference in remuneration grades in the NHS. Cost savings would be more prominent in units where consultants regularly deliver SLT. To date there are no published data on the cost savings of an optometrist-delivered SLT service, but SLT has been shown to be more cost-effective than medical treatment when performed by consultant ophthalmologists, allowing for five additional ophthalmology specialist appointments for every patient given SLT [1].

The National Institute for Health and Care Excellence (NICE) has reported recent concerns of topic experts around an upcoming ‘considerable resourcing issue’ for SLT and a need to train non-medical professionals for SLT delivery [11]. The perceived stability of the optometric workforce is expected to result in a stable optometrist-delivered SLT service, independent of the availability and rotations of ophthalmologists. Continuity of care could be improved, reducing medical costs, service use and complications [15, 16], whilst being welcomed by patients [17].

An optometrist-delivered SLT service would also support the most appropriate utilisation of senior ophthalmologists’ time. Approximately 20% of current glaucoma service workload requires specialist input and, although consultant-led clinics currently focus on high risk cases, the Royal College of Ophthalmologists calls for a service restructure [10, 18]. The number of glaucoma patients is expected to grow, but with nearly 30% of senior ophthalmologists nearing retirement current backlogs are expected to increase [10, 19]. Delivery of SLT by optometrists would allow consultants more time to attend to complex and/or surgical cases, a skill not mastered by others in the HES.

### Training

Experience in gonioscopy was considered necessary for the safe delivery of SLT. Optometrists were expected to make a judgement of the angle of the patients, instead of performing SLT as ‘instructed’, supporting their expected independence. Independent prescribing rights were anticipated to minimise the need for medical input around discharge medication and the management of post-SLT complications and subsequently improve service flow.

Higher qualifications in glaucoma, available by the College of Optometrists, were not considered necessary, but useful, for optometrists wishing to train for SLT. Among the three available qualifications, the Higher Certificate in glaucoma was considered the most appropriate. Optometrists with higher qualifications or experience in specialist HES clinics show safe clinical decision making in glaucoma management, comparable to consultant ophthalmologists [20–22], and perform better than optometrists without such qualifications or experience [23].

Clinicians expected the training to maintain the existing apprenticeship format [4, 5]; optometrists would undergo theoretical clinical and laser safety training, followed by a period of observing, delivering SLT under supervision and then being signed off as competent. Chadwick et al. [4] reported a minimum of five supervised procedures, similar to the Moorfields protocol for non-medical SLT training [24], but this was often exceeded, mainly due to a lack of adequate challenging cases or self-confidence [4]. In agreement with the literature, clinicians interviewed for this study identified that practical training should follow a skills based approach. The current lack of training standardisation [3, 4, 24] may be linked to difficulties in ensuring an adequate case mix and the subsequent delay in clinical readiness.

### NHS infrastructure and support

Optometrists wishing to train for SLT were expected to have experience of a consultant-led glaucoma clinic. The structured progression within the NHS was associated with a perceived seniority; optometrists are first introduced to optometrist-led glaucoma clinics and then progress to a consultant-led clinic, with a more complicated case mix. Consultant-led clinics were also perceived to allow interaction and mentorship between optometrists and ophthalmologists for the discussion of case management and technique. The latter did not invalidate the independence of the optometrists; reports by optometrists performing SLT are supported by the literature and indicate that although the frequency of case discussion with ophthalmologists varies between clinicians, urgent ophthalmologist intervention has not been required [4]. Patients described feeling reassured by the availability of ophthalmologists in the HES, which was reflected in their concerns around community optometrist-delivered SLT.

Clinicians also felt that optometrist-delivered SLT is best performed in the HES, ensuring appropriate clinical support. Some acceptance of a community scheme was reported, provided that the treatment decision was made in the HES and that there was documented maintenance of the necessary clinical skills and/or a regular clinical role in the HES. This is in agreement with previous research, indicating that clinical decision making is improved in optometrists with HES experience [20–23].

### Challenges

One of the perceived challenges related to the current lack of standardised training and governance. To date, there is no official qualification or competency framework for non-medical professionals wishing to deliver SLT in the HES. The Common Competency Framework [25] supports the development of training and accreditation for clinicians of varied professional backgrounds and the College of Optometrists higher qualifications enable optometrists to participate in HES glaucoma clinics and manage glaucoma patients [25, 26] according to current NICE guidelines (NG81) [27]; none of the above, however, specify the required training for SLT delivery. The lack of formal training also reflected on the governance of such procedures. To date, optometrists performing laser procedures in the HES are covered by their indemnity insurance provider, under the assumption that they have been adequately trained, accredited and that they practice according to local protocols. A successful rollout of an optometrist-delivered service in the HES, should be preceded by the drafting of national standard training and treatment protocols.

Previous research indicates that the current apprenticeship-format SLT training for optometrists in the UK may prove insufficient for some, due to a limited case mix or a lack of confidence [4]. A standardised training framework, transferrable between units and professional groups may prove beneficial for the national rollout of the service, whilst maximising patient quality of care, supporting professional best practice and assisting compliance with litigation standards. The collaboration between professionals and the impact of optometrists’ SLT training on ophthalmologists’ training were also perceived as challenges by clinicians, but it was concluded that ophthalmology training could remain unaffected if careful planning and inter-disciplinary training were achieved.

Ophthalmologists in the UK are currently required to have performed 30 IOP-reducing procedures, including laser treatments, upon completion of training [28]; these may include SLT, peripheral iridotomy, argon laser trabeculoplasty, iridoplasty and cyclodiode, leading to a possible variability in the type of laser procedures and the number of cases amongst graduating ophthalmologists. Current optometrist training SLT protocols dictate the delivery of at least five SLT treatments under supervision [24] and there is no indication that, once gonioscopy and prescribing rights are mastered, optometrists’ SLT training needs are different to those of ophthalmologists. There are also no technical specifications on the training of ophthalmologists or optometrists in SLT; the Royal College of Ophthalmologists Ophthalmic Specialist Training guide states that trainees should be encouraged to practice on model eyes before performing common laser procedures, but the use of a sidearm or video slit lamp is only considered necessary for laser photocoagulation. The length and extent of training requirements and the potential benefits of a progressive or rapid approach to skill acquisition, should also be considered when developing or revising training programmes for any professionals in new procedures.

Participants in this study indicated that ophthalmologists’ training could benefit from the development of an optometrist-delivered SLT service, via the utilisation of a common, formal, structured training framework and by inter-disciplinary training. The identified pre-requisites for optometrists could be used for the training of ophthalmologists as well, to ensure the smooth running of the training process for any professionals involved, whilst technical and time-based specifications of the training and the exact form of supervision should be clarified for any professionals wishing to perform SLT.

### Ophthalmic nurse practitioners

This study focussed on an optometrist-delivered SLT service, but useful information around the utilisation of ophthalmic nurses for the delivery of SLT has emerged. Nurse practitioners have an active role in glaucoma virtual clinics and patient education [18, 29] and are accepted in advanced roles [30]. However, clinicians and patients showed reluctance towards a nurse-delivered SLT service at this initial stage. This was related to a perceived steeper learning curve and increased training needs, confirming previous work by the Royal College of Ophthalmologists [10]. This study identified the necessity of skills not commonly mastered by nurse practitioners, such as independent management of glaucoma, prescribing and gonioscopy. To date there is a single report of agreement in gonioscopy between a single nurse practitioner and a consultant ophthalmologist, in a limited case mix of open angles [31].

## Conclusion

This is the first study to address the views of ophthalmologists, optometrists, managers and patients around an optometrist-delivered SLT service in the UK HES. Optometrist-delivered SLT has been a topic of discussion and professional development events over the past year, yet there is lack of a standardised training scheme and governance. The study adopted a qualitative approach, highlighting a number of previously not considered themes that should be taken into account when planning these services, e.g. pre-requisites for training/clinical experience, patient acceptability, impact on ophthalmology training and community delivery of SLT.

Recruitment of patients aimed at a representative sample of the current model of SLT delivery at MEH, where NHS SLT clinics are equally split between ophthalmologists and optometrists. Patients recruited from the LiGHT trial cohort were predominantly treated by an ophthalmologist, whilst patients recruited from the NHS clinics were mainly treated by an optometrist. Clinicians and managers were members of staff at Moorfields Eye Hospital. Although both these professional groups related to units with different needs based on their overall or previous professional experience and judgement, it is possible that the views expressed relate to the nature and volume of services provided at Moorfields, as well as a possibly stronger support for SLT and/or optometry enhanced roles and may not be representative of clinicians and commissioners nationwide. Nevertheless, the reported findings would still apply to units with a similar demand and breadth of services to Moorfields Eye Hospital.

The development and rollout of an optometrist-delivered SLT service seems an important step towards addressing the current and expected demand for SLT; the European Glaucoma Society [32] and the American Academy of Ophthalmology [33] now recommend the use of SLT as initial treatment for open angle glaucoma, whilst the UK-based NICE is anticipated to issue relevant updated guidelines [11]. Careful design of a standardised, transferrable training scheme and robust governance are necessary for the national rollout of optometrist-delivered SLT. Patients appear positive to this change and acknowledge the benefits to the NHS and the clinical services.

## Summary

### What was known before

- SLT is an effective IOP lowering procedure, now recommended as a 1st line treatment in the EU and the USA.
- Delivery of SLT by optometrists is happening in an ad-hoc basis in the UK hospital eye service.
- Evidence on training requirements, service efficacy, clinical safety or cost-effectiveness is limited.

### What this study adds

- Gonioscopy and independent prescribing rights, were perceived as necessary for optometrists undertaking SLT training.
- An optometrist-delivered SLT service was expected to benefit the NHS.
- Patients were accepting of an optometrist-delivered SLT service in the hospital eye service.

## Supplementary information

Appendix 1 - COREQ checklist

Appendix 2 - Participant quotations
