# Correction to: Selective laser trabeculoplasty (SLT) performed by optometrists —enablers and barriers to a shift in service delivery

**DOI:** 10.1038/s41433-021-01865-8

**Published:** 2021-12-20

**Authors:** Evgenia Konstantakopoulou, Lee Jones, Neil Nathwani, Gus Gazzard

**Affiliations:** 1 NIHR Biomedical Research Centre at Moorfields Eye Hospital NHS Foundation Trust, London, UK; 2 Institute of Ophthalmology, University College London, London, UK; 3 Division of Optics and Optometry, University of West Attica, Athens, Greece

**Keywords:** Health services, Glaucoma

Correction to: *Eye* 10.1038/s41433-021-01746-0, published online 13 August 2021

Unfortunately, a comment from the author to our vendor was published in the part Data collection.

We apologize for this mistake.

The original article has been corrected.

