# Supplementary material for: Selective laser trabeculoplasty (SLT) performed by optometrists—enablers and barriers to a shift in service delivery
Source: Eye (Lond). 2021 Aug 13;36(10):2006–12. doi: 10.1038/s41433-021-01746-0 (PMC8362647; doi:10.1038/s41433-021-01746-0)
Supplement: Supplementary file 2 — Appendix 2 - Participant quotations [file 41433_2021_1746_MOESM2_ESM.pdf]

Key theme	Quotation
**Clinical practice & training**	
Non-medical professionals	*Optometrists are naturally in a position where they're going to be learning skills like gonioscopy and slit lamp; it's part of the core training to handle glaucoma. So, it seems to be a more natural step for them than for others. (Consultant)*
	The optometrist that we use as a family is actually very good and they're the ones that found that I had glaucoma. I feel fairly confident that they could do the work if they were trained. (Patient)
Professional activity prior to and around SLT training	*I've been doing SLT for many years. On a six-month basis I might have a chat with the consultant next door regarding a patient. (Optometrist)*
Skills prior to SLT training	*They would need to be independent prescribers to really know about managing someone post-SLT. (Fellow)*
SLT training	*The priority is clear-cut training schemes that are accepted by the Colleges of Optometrists and Ophthalmologists. (Manager & Consultant)*
**Advantages**	
Capacity & reduced waiting times	*If we have a service that is supposed to just be doing SLT, patients can be booked in with much more control and tighter schedule to ensure they don't overlap. I think that would make the whole management of clinics easier. (Fellow)*
	It would allow hospital services to meet the demand that has resulted from favourable results from the LiGHT Trial. I think we would struggle if we didn't look at using allied healthcare professionals as well. (Fellow)
Costs	*It is certainly a lower remunerated session than a consultant or an associate specialist. But this might be offset by the number of cases an optometrist will do in a session versus a consultant. (Consultant)*
	It is certainly a lower remunerated session than a consultant or an associate specialist. But this might be offset by the number of cases an optometrist will do in a session versus a consultant. (Consultant)
Optometrist availability/stability/manpower	*We'd see a more stable workforce. Trainees and fellows cycle through the department rapidly; optometrists are often more long-term. (Fellow)*
	I've lasered a few patients three times or four times now; the continuity is important and I think patients like that in the NHS setting. (Optometrist)
**Disadvantages & concerns**	
Time commitments	*Optometrists may drop something else that they're doing to incorporate the laser clinical role, which means that we have to recruit for those positions. That's not so much of an issue because there's plenty of people that want to do hospital work. (Manager & Optometrist)*
Governance & litigation	*I'd say the risk to the organisation is greater, but providing there's a governance framework and training is*

	to a good level that will mitigate much of that risk. (Consultant)
**Challenges**	
Professional boundaries & interactions	*Anxiety may be produced by an apparent blurring of professional boundaries within the professions. The institutions may face resistance from a few ophthalmologists who are not comfortable with the expansion of the optometrists' role. (Manager & Consultant)*
**Impact on ophthalmology training**	
	As long as we have made some provisions for training of the juniors, I don't see why it should impact negatively. (Consultant)<br>*In a small department there may be only a couple of consultant ophthalmologists. If you have a dedicated optometrist doing all the lasers, then certainly the juniors, the SpRs, will have limited exposure to doing enough cases to become proficient themselves. (Optometrist)*
**SLT in the community**	
	I think the optometrist who works in the community and wants to deliver SLT should retain that link with hospital care, in a consultant face to face clinic. (Consultant)<br>*Once the decision for treatment is made then the laser itself could be done by anyone, anywhere, under the appropriate governance framework, provided the competency has been gained and the audit process affirms that the individual is achieving results. (Consultant)*<br>*Because it's private sector, as a customer, you don’t have any idea of what the regulation is, the governance, central supervision of standards. I would not have been comfortable for something which you perceive as being invasive. (Patient)*
**Patient expectations & values**	
	What matters most to me is that I understand what their (the optometrist’s) credentials are, to have done it, to be doing it and that I trust them. (Patient)<br>*Their communication skills, being very assured and friendly and having good listening and intercommunication skills. (Patient)*<br>*The person delivering the treatment needs to be qualified, experienced and then have interpersonal skills to explain what’s going on. (Patient)*
**Other health-care professionals**	
	Nurses would need more training; they are not trained in any of the angle structures. For the time being I would go for the optometrists in the first instance. (Fellow)<br>*There would be some nurse practitioners that would be very well placed to do this if they were used to seeing and treating glaucoma patients already. (Fellow)*<br>*I'm not particularly happy about a nurse. It depends - is this nurse specialising in eyes? An optometrist may*

	not be medically trained, but eyes are their business. (Patient)<br>*I don't particularly see something that's going to affect the ophthalmologists' training; if a junior doctor wants to train I would rather support them, rather than me trying to take over the patient. (Nurse)*
